# Xiaoyaosan Improves Antibiotic-Induced Depressive-Like and Anxiety-Like Behavior in Mice Through Modulating the Gut Microbiota and Regulating the NLRP3 Inflammasome in the Colon

**DOI:** 10.3389/fphar.2021.619103

**Published:** 2021-04-16

**Authors:** Wenzhi Hao, Jiajia Wu, Naijun Yuan, Lian Gong, Junqing Huang, Qingyu Ma, Huizheng Zhu, Hua Gan, Xiaoli Da, Lijuan Deng, Xiaojuan Li, Jiaxu Chen

**Affiliations:** ^1^ Guangzhou Key Laboratory of Formula-Pattern of Traditional Chinese Medicine, Formula-Pattern Research Center, School of Traditional Chinese Medicine, Jinan University, Guangzhou, China; ^2^ School of Basic Medical Science, Hubei University of Chinese Medicine, Wuhan, China; ^3^ School of Traditional Chinese Medicine, Beijing University of Chinese Medicine, Beijing, China

**Keywords:** Xiaoyaosan, antidepressant, anxiolytic, lipopolysaccharide, gut microbiota

## Abstract

Disturbance of the gut microbiota plays an essential role in mental disorders such as depression and anxiety. Xiaoyaosan, a traditional Chinese medicine formula, has a wide therapeutic spectrum and is used especially in the management of depression and anxiety. In this study, we used an antibiotic-induced microbiome-depleted (AIMD) mouse model to determine the possible relationship between imbalance of the intestinal flora and behavioral abnormalities in rodents. We explored the regulatory effect of Xiaoyaosan on the intestinal flora and attempted to elucidate the potential mechanism of behavioral improvement. We screened NLRP3, ASC, and CASPASE-1 as target genes based on the changes in gut microbiota and explored the effect of Xiaoyaosan on the colonic NLRP3 pathway. After Xiaoyaosan intervention, AIMD mice showed a change in body weight and an improvement in depressive and anxious behaviors. Moreover, the gut flora diversity was significantly improved. Xiaoyaosan increased the abundance of Lachnospiraceae in AIMD mice and decreased that of Bacteroidaceae, the main lipopolysaccharide (LPS)-producing bacteria, resulting in decreased levels of LPS in feces, blood, and colon tissue. Moreover, serum levels of the inflammatory factor, IL-1β, and the levels of NLRP3, ASC, and CASPASE-1 mRNA and DNA in the colon were significantly reduced. Therefore, Xiaoyaosan may alleviate anxiety and depression by modulating the gut microbiota, correcting excessive LPS release, and inhibiting the immoderate activation of the NLRP3 inflammasome in the colon.

## Introduction

Depression and anxiety are the most common psychiatric disorders, affecting more than 350 million people globally (Baxter et al., 2014). These mental disorders can cause significant impairment, increase annual care costs, and represent a significant economic burden worldwide (Kessler and Bromet, 2013). Current drug treatments for these diseases are limited and cause many toxic side effects (Rieder et al., 2017). Extensive evidence indicates that intestinal flora plays an essential role in the pathophysiology of depression and anxiety (Sherwin et al., 2018). Changes in the gut microbiota have been reported in patients with depressive disorder and anxiety (Chen et al., 2018), and similar results have been observed in rodent models with these conditions (Hyo-Min et al., 2018). Probiotic treatment can improve the clinical symptoms of patients with depression and anxiety (Slykerman et al., 2017). These findings indicate that gut microbes are involved in the development of depression and anxiety and could be a potential target for drug development in the management of such conditions. However, the exact role of the intestinal flora in the pathogenesis of depression and anxiety is unknown and the downstream physiological mechanisms by which gut microbes influence human behavior remain unclear (Sender et al., 2016). Recent findings provide new insights into the pathogenesis of depression and anxiety mediated by intestinal flora, suggesting that the effects of gut microbiota may be associated with the NLRP3 inflammasome.

The NLRP3 inflammasome is a multiple protein complex consisting of nod-like receptor protein 3, adaptor protein ASC, and procaspase-1 precursor that was discovered and reported in 2002 (Martinon et al., 2002). It has been reported that the NLRP3 inflammasome can recognize the invasion of internal and external pathogens. Targeting NRLP3, as a therapeutic approach, results in activating caspase-1 and promotes the maturation and secretion of pro-IL-1β and pro-IL-18, thus triggering the anti-pathogenic immune-inflammatory response of the body (Du et al., 2016). The NLRP3 inflammasome can be activated by several agents including bacteria, fungi, viral components, extracellular ATP, and silica crystals (Jo et al., 2015). Moderate activation of the NLRP3 inflammasome plays a role in several diseases including Alzheimer’s disease, anxiety, and depression (He et al., 2019). Current evidence has promulgated the link between the NLRP3 inflammasome and gut microbes, indicating that changes in intestinal flora can impact the identification and response of inflammatory sensors and further regulate the activation of the NLRP3 inflammasome (Pérez-Figueroa et al., 2015).

Xiaoyaosan, a compound formula in traditional Chinese medicine (TCM), comprises eight crude herbs including *Bupleurum chinense DC, Paeonia lactiflora Pall, Angelica sinensis (Oliv.) Diels, Atractylodes lancea (Thunb.) DC, Wolfiporia extensa (Peck) Ginns (syn. Poria cocos (Schwein.) F.A.Wolf), Glycyrrhiza glabra L., Mentha canadensis L.,* and *Zingiber officinale Roscoe* in a 5:5:5:5:5:4:1:5 ratio. This formulation was first described in a medical book, Taiping Huimin Heji Jufang, which was written during the Song Dynasty of China (960-1127 AD) (Li et al., 2017). Xiaoyaosan has been regarded as a remedy for liver-qi stagnation and spleen deficiency, while modern pharmacological studies have proven its efficacy in treating anxiety and depression (Zhang et al., 2012). Previous studies have suggested that Xiaoyaosan can improve depressive behavior in the CRS rat-model by regulation of the intestinal flora (Zhu et al., 2020). However, the specific mechanism by which Xiaoyaosan improves depressive behavior by modulating the intestinal flora and whether colon inflammation and NLRP3 inflammasome are related to the antidepressant mechanism of Xiaoyaosan need to be further studied. In this study, we aimed to investigate further whether Xiaoyaosan exerted antidepressant effects by modulating the gut microbiota and restraining the immoderate activation of the NLRP3 inflammasome in the colon.

## Materials and Methods

### Animals

Sixty healthy 8-week-old male C57BL/6 SPF mice [SYXK (Yue) 2017-0174] weighing 20 ± 2 g were purchased in advance, and they were subjected to a 7-days adaptive feeding process before the start of the formal experiment. The experimental conditions were strictly implemented and included room temperature of 21 ± 2°C, relative humidity 30–40%, and 12 h light/12 h dark cycle. The animal experiments were reviewed by the Animal Ethics Committee (IACUC-20200810-04) and all experiments complied with the current animal-welfare guidelines.

### Behavioral Procedures

**FIGURE 1:**
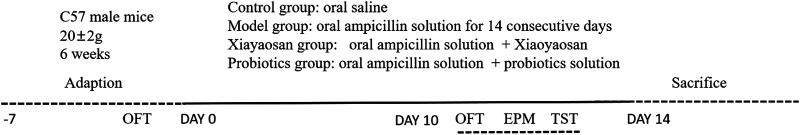
Experimental flowchart. Mice were divided into four groups: the control group, model group, Xiaoyaosan treatment group, and probiotics treatment group. The model group received oral ampicillin solution for 14 consecutive days at a dose of 100 mg/kg and an intragastric volume of 0.1 ml/10 g body weight. The control group received oral saline in an equivalent volume and served as a negative control. Mice in the two treatment groups received Xiaoyaosan (0.658 g/kg/d) and probiotics solution (0.15 ml/d), respectively.

Mice were randomly divided into four groups as follows: control, model, Xiaoyaosan, and probiotics. The model group was administered ampicillin (100 mg/kg and 0.1 ml/10 g body weight) in freshly diluted phosphate-buffered saline (PBS; pH 7.2), once daily for 14 consecutive days (Hyo-Min et al., 2018). The control group received an equivalent volume of saline and served as a negative control. The mice in the Xiaoyaosan group and the probiotic group received Xiaoyaosan (0.658 g/kg/d) and probiotics solution (0.15 ml/d) containing *Bifidobacterium longum, Lactobacillus acidophilus, Bifidobacterium bifidum, Bifidobacterium breve, Bifidobacterium lactis, Lactobacillus brevis, Lactobacillus bulgaricus, Lactobacillus casei, Lactobacillus helveticus, Lactobacillus plantarum, Lactobacillus reuteri, Lactobacillus rhamnosus, Lactobacillus salivarius, Lactococcus lactis, Streptococcus thermophilus, and Bifidobacterium infantis* (Figure 1). The weight of mice in each group was recorded one day before the start of the experiment on day 0, and then on days 7 and 14 of the experiment, for comparison.

#### Open Field Test

The classic “open field” behavioral test method is mainly used to observe autonomous behavior, exploratory behavior, and stress of experimental animals in an unaccustomed environment. OFT was performed on days 0 and 14 in our study. Mice were placed in a behavioral operating room for 10 min for adaptation and then moved to the center zone. Camera recording was initiated and timed, and the behavior of the mice was observed for 5 min. Immediately after each experiment, the boxes were cleaned with 75% alcohol. OFT was performed using the internationally recognized Behavior Analysis software (EthoVision software analysis system, Noldus Information Technology) and included the analysis of the total movement distance of each group of mice (Choleris, 2001).

#### Tail Suspension Test

Mice were suspended on a horizontal rod 50 cm from the ground, with the tails fixed using adhesive tape. The entire experimental period lasted for 6 min. The activity of each mouse for the last 4 min was recorded. Immobility time (seconds) was defined as the time required by the mice to give up struggling and remain completely motionless (Steru et al., 1985). The TST experiment data were analyzed using the Behavior Analysis software.

#### Elevated Plus-Maze Test

The EPM experiment was performed using a plus-maze device consisting of two open arms, two closed arms, and a central platform. The dimensions of the open and closed arms were both 30 × 7 cm, with the closed arms being covered by 20-cm baffles on both sides. The mice were placed individually in the central area (7 × 7 cm) at 60 cm above the ground, and their movements were recorded for 5 min (Rodgers and Johnson, 1995). Results of the EPM test were analyzed using the EthoVision software analysis system (Noldus Information Technology) and included the analysis of animal movement distance in open arms, duration time, and percentage of open arm duration time.

### Sample Collection and Preparation

After the last behavioral test was completed, the mice were sacrificed using intraperitoneal injection of 3% sodium pentobarbital (100 mg/kg), and the feces were collected from the cecum in a sterile environment. The fecal samples were immediately stored at 20°C until DNA extraction. Colon tissues of mice were collected and stored for western blotting and qRT-PCR testing. The remaining colon tissues were stored in 4% paraformaldehyde solution at 4°C and subsequently used for tissue sectioning and immunohistochemistry.

#### 16S Microbial Diversity Analysis

The E.Z.N.A.^®^ Soil DNA Kit (Omega Bio-Tek, Norcross, GA, United States) was used to separate and extract the microbial DNA from the stool sample. The V3–V4 hypervariable region of the bacterial 16S rRNA gene was amplified with the primers 338F (5′-ACT​CCT​ACG​GGA​GGC​AGC​AG-3′) and 806R (5′-GGACTACHVGGGTWTCTAAT-3′). The PCR program was completed using a thermal cycling PCR system (GeneAmp 9700, ABI, United States) using the following program: denaturation at 95°C for 3 min; at 95°C for 27 cycles of 30 s; annealing at 55°C for 30 s; 45 s for an extension at 72°C; and final extension at 72°C for 10 min. Trimmomatic was used to filter the quality of the original fastq files and merge the data using FLASH. The similarity of taxonomic units (OTUs) was classified using UPARSE (version 7.1, http://drive5.com/uparse/) according to 97% criterion, and UCHIME was used to identify and remove chimeric sequences. Lastly, the RDP classifier algorithm (http://rdp.cme.msu.edu/) program was executed on the Silva (SSU123) 16S rRNA database using a 70% confidence threshold to analyze the classification of each 16S RNA gene sequence.

### Bioassays

#### H&E Staining

Samples of colonic tissue were fixed in 10% formalin, decalcified, dehydrated, made transparent, and then dipped and embedded in paraffin. Next, 5-µm-thick tissue samples were prepared using a microtome. Sections were dewaxed with xylene, passed through an aqueous ethanol series, stained using HE, and observed using microscopy.

#### ELISA

Serum LPS and IL-1β levels were determined using an ELISA detection kit (Cusabio Biotech., LTD., Wuhan, China). LPS levels in the colon homogenate and fecal supernatant were determined. LPS and IL-1β levels were determined by measuring the absorbance using a microplate reader and plotting a standard curve.

#### Quantitative Real-Time PCR Analysis

TRIzol reagent (Applied Biosystems, Waltham, MA, United States) was used to extract total RNA, and cDNA was synthesized using the RevertAid First Strand cDNA Synthesis Kit (Thermo Fisher Scientific, Waltham, MA, United States). The primers for NLRP3, ASC, and CASPASE-1 mRNA sequence used were as described in NCBI (shown in Table 1) and were synthesized by Sangon Biotech Co., Ltd (Shanghai, China). SYBR®Green PCR Master Mix (Applied Biosystems) was used as the qRT-PCR reaction system with a volume of 25 µl and detected using a Multicolor Real-time PCR detection system (Bio-Rad, Hercules, CA, United States). The testing conditions were as follows: 95°C for 10 min, followed by 40 cycles of 95°C for 30 s and 60°C for 1 min. The 2^−ΔΔCt^ method was used to calculate relative mRNA expression in each sample.

**TABLE 1:** Primer sequences used for qRT-PCR.

Gene	Forward primer (5,–3)	Reverse primer (5,–3)
NLRP3	GAG​CTG​GAC​CTC​AGT​GAC​AAT​GC	ACC​AAT​GCG​AGA​TCC​TGA​CAA​CAC
ASC	GAA​GTG​GAC​GGA​GTG​CTG​GAT​G	CTT​GTC​TTG​GCT​GGT​GGT​CTC​TG
CASPASE-1	ACA​ACC​ACT​CGT​ACA​CGT​CTT​GC	CCA​GAT​CCT​CCA​GCA​GCA​ACT​TC
GAPDH	TGA​AGG​GTG​GAG​CCA​AAA​G	AGT​CTT​CTG​GGT​GGC​AGT​GAT

#### Immunohistochemical Staining of Colon Tissues

Immunohistochemical staining was used to characterize the paraffin-embedded colon tissues. After washing, heating, dewaxing, dehydration, and antigen retrieval, the sections were treated with primary antibodies (NLRP3, Abcam, 1:150; ASC, Santa, 1:150; Caspase-1, Abcam, 1:10) at 37°C for 1 h and then at 4°C overnight. The slides were then incubated with the corresponding secondary antibodies (anti-mouse, Servicebio, United States; anti-rabbit, Servicebio, United States) at room temperature. The sections were developed using DAB (3,3-diaminobenzidine) reagent (Invitrogen, Carlsbad, CA, United States). After washing with PBS, the sections were re-stained with hematoxylin, dehydrated, and then photographed and recorded at ×400 magnification (Leica, Leica Co., Tokyo, Japan). Mean Optical Density (MOD) was calculated using Image-Pro Plus software (version 6.0; Media Cybernetics, Bethesda, MD, United States).

### Western Blotting of Colon Tissue

WB was used to determine the expression of NLRP3, ASC, and CASPASE-1 proteins in the colon tissue. A tissue protein extraction kit was used to extract total protein from the colon of each mouse and perform the BCA (Beyotime) protein assay. After electrophoresis and electroporation, the converted membrane was blocked with 5% skimmed milk powder for 1 h, washed with TBST buffer, and incubated with the primary antibody overnight at 4°C. The electrophoretic membrane was then incubated with a secondary antibody for 1 h and washed with TBST buffer; luminescence was measured using a chemiluminescence reagent (Millipore, Billerica, MA, United States). The protein-separation membrane was scanned and analyzed using an image analyzer (Bio-Rad, California, United States).

### Statistical Analysis

Data are expressed as mean ± SD and tested for normality and homogeneity of variance by using the SPSS25.0 software (Chicago, IL, United States). Data produced by repeated measurements were first analyzed via repeated analysis of variance (ANOVA), if the data were normally distributed and homogenous. If the data were not normally distributed or the variance was not uniform, the non-parametric test of K independent samples was used for item-by-item statistical analysis. A *p*-value < 0.05 indicated significant differences for all the statistical tests. Drawing was performed using the GraphPad Prism 7.0 software (La Jolla, United States).

## Results

### Quality Control of Xiaoyaosan by UPLC-Q-TOF/MS

Refer to the previous literature, the quality control of Xiaoyaosan was investigated by UPLC-Q-TOF/MS.(Xiaoyaosan is purchased from Jiuzhitang Group Co. Ltd., it has come from the same preparation and has the same batch number:20190724). The UPLC-Q-TOF/MS chromatogram of Xiaoyaosan is shown in Supplementary Figure S1A. As shown in Supplementary Figure S1B, seven compounds were distinguished: 1. Paeoniflorin; 2. Kaempferol; 3. Quercetin; 4. Aloe emodin; 5. Luteolin; 6. Glyasperin C; 7. Acacetin.(Yuan et al., 2020).

### Effect of XYS on the Body Weight of Antibiotic-Induced Mice

**FIGURE 2:**
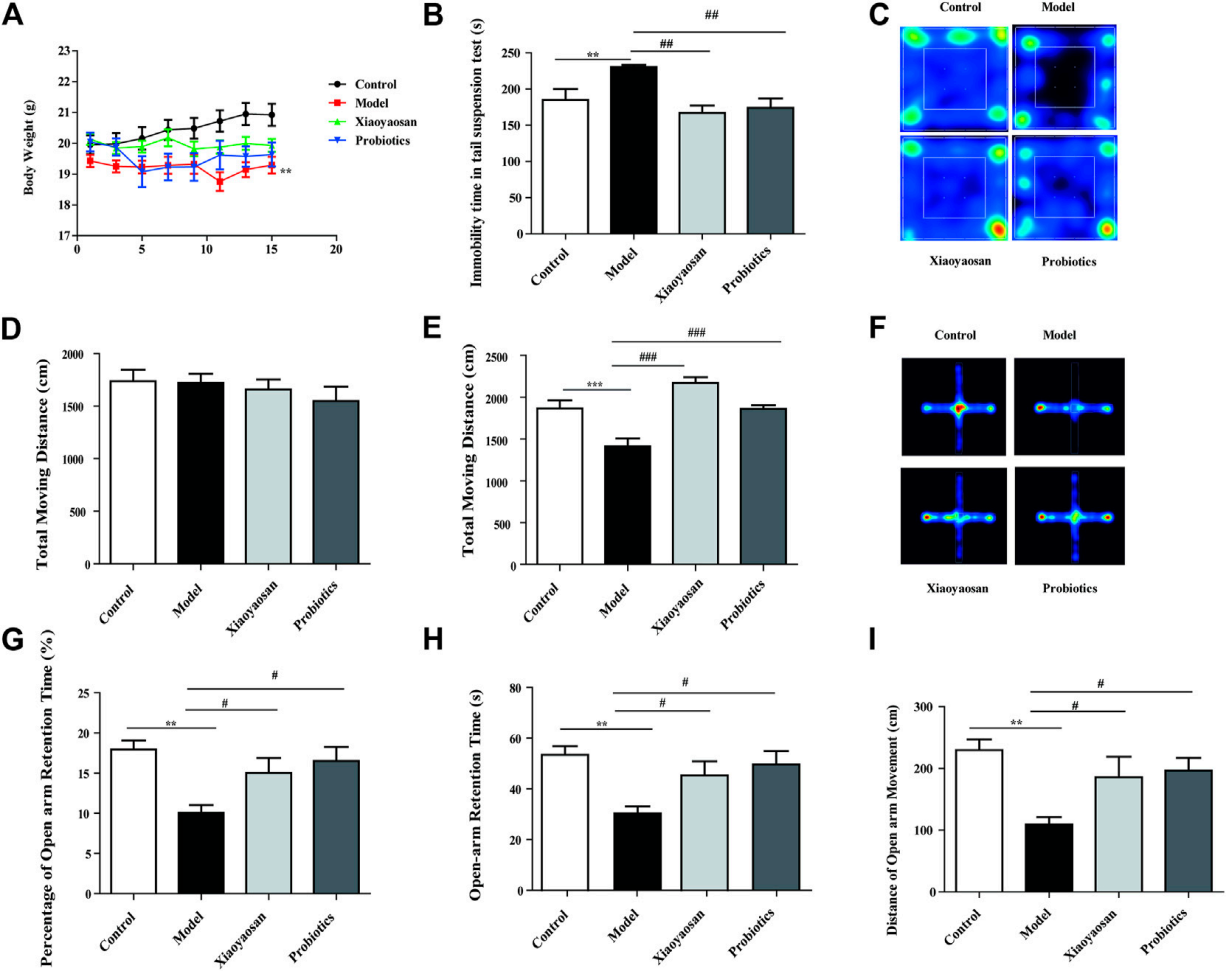
Xiaoyaosan improves depressive-like behavior and anxiety-like behavior in antibiotic-induced mice. **(A)** Changes in body weight. **(B)** TST. **(C)** Heat map of OFT. **(D)** OFT on day 0. **(E)** OFT on day 14. **(F)** Heat map of FPM. **(G, H, I)** EPM. Data are expressed as the mean ± SEM. **p < 0.01, ***p < 0.001 vs. the control group; ^#^ *p* < 0.05, ^##^ *p* < 0.01, ^###^ *p* < 0.001 vs. the antibiotic-induced model group. *n* = 10 per group.

On day 0 and day 7, there was no statistical difference in body weight across the four groups. However, on day 14, the weight of antibiotic-induced mice group decreased significantly in comparison with that observed in the normal group (*p* < 0.01). Moreover, the body weight of the Xiaoyaosan group and probiotics group was higher than that of model group, but the difference was not significant (Figure 2A). The results suggested that the weight of the model group was significantly reduced after 14 days of antibiotic intervention while treatment with XYS or probiotics could resist antibiotic-induced weight loss.

### Effect of XYS on Antibiotic-Induced Depressive-Like Behavior in Mice

To examine the effects of Xiaoyaosan on depressive-like behaviours, several behavioral tests including the tail suspension test (TST) and the open field test (OFT) were conducted.

For the TST test, antibiotic-induced mice showed increased immobility time in comparison with control mice (*p* < 0.01), recorded as the absence of escape-oriented behaviour in the TST (Figure 2B). For mice treated with Xiaoyaosan and probiotics, the immobility time in the experiment was remarkably reduced vs. the model group (*p* < 0.01).

For the OFT illustrated in Figure 2C, the heat map data shows the movement status of mice in open field experiments. As shown in Figure 2D, on day 0, the total distance travelled during 5 min was not significantly different when compared across the four groups. However, as shown in Figure 2E, on day 14, the antibiotic-induced mice displayed a significant decrease in the total distance in comparison with the normal group (*p* < 0.001), suggesting that the model group was experiencing obvious depressive-like behavior. However, this change was effectively improved by treatment with Xiaoyaosan (*p* < 0.001) and probiotics (*p* < 0.001).

### Effect of XYS on Antibiotic-Induced Anxiety-Like Behavior in Mice

To examine the effects of Xiaoyaosan on anxiety-like behaviors, elevated plus-maze test (EPM) was conducted.

For the EPM illustrated in Figure 2F, the heat map data shows the movement status of mice in elevated plus-maze test. As illustrated in Figure 2G, in the EPM experiment, the percentage of time spent in open arms was significantly less in the model group than in the control group (*p* < 0.01). The percentage of time spent in open arms of Xiaoyaosan-treated animals was higher than in model, confirming the reduction in anxiety-like behaviors in rodents. In addition, the same results were obtained for the time spent in the open arms (Figure 2H). In line with the above results, an obvious decrease in the distance travelled in the open arms (Figure 2I) was observed in the antibiotic-induced group vs. the control group. The results of the heat map (Figure 2B) also showed that the range of movement of antibiotic-induced mice was mainly concentrated in the closed arm, while the range of movement of Xiaoyaosan and probiotics was explored toward the open arm.

### Effect of XYS on Gut Microbiota of Antibiotic-Induced Mice

**FIGURE 3:**
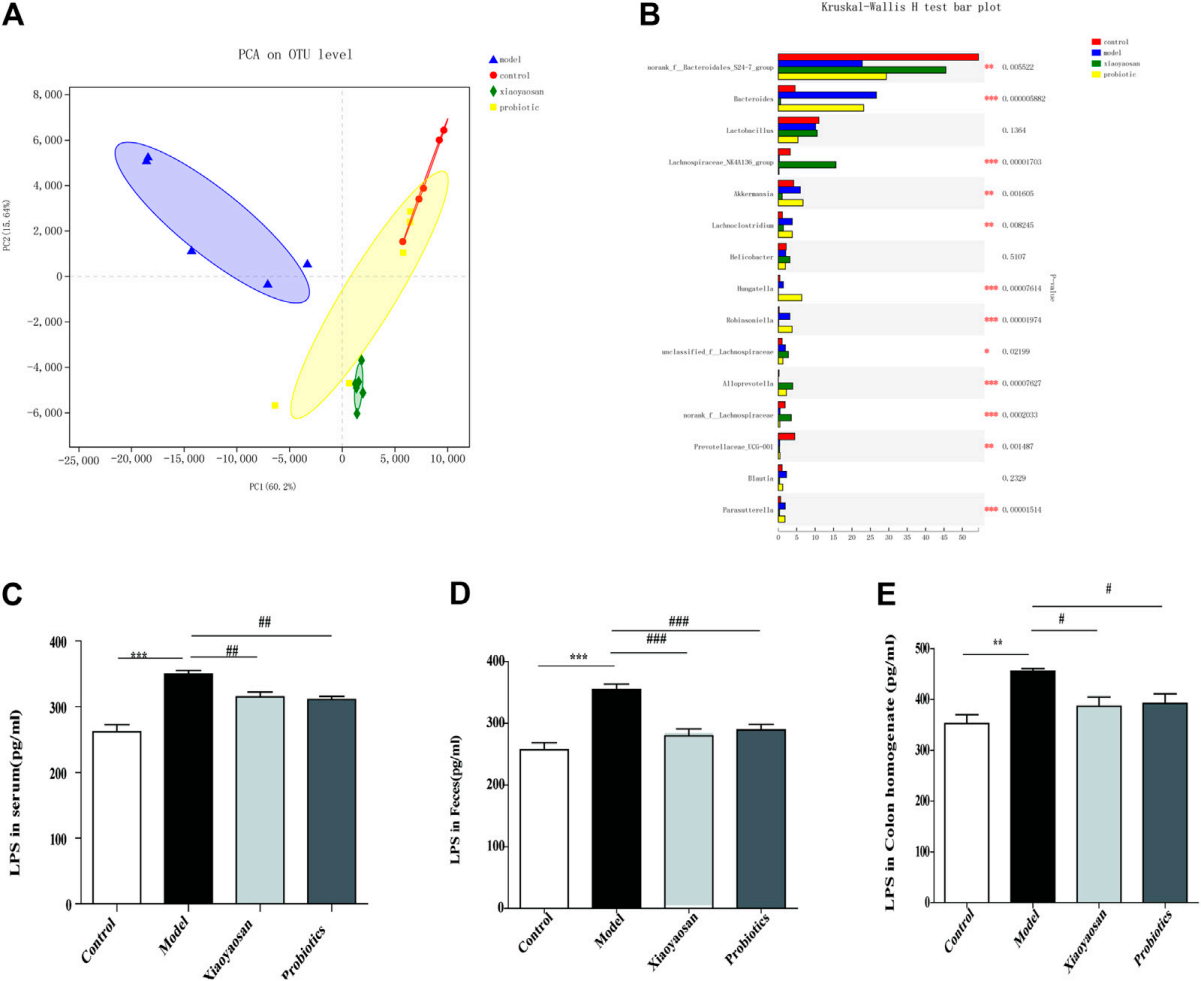
Effect of XYS on gut microbiota and LPS Level of antibiotic-induced mice. **(A)** PCA principal component analysis. **(B)** Results of a completely randomized multi-sample rank sum test. **(C)** Effects of Xiaoyaosan on LPS Level of Serum. **(D)** Effects of Xiaoyaosan on LPS Level of feces. **(E)** Effects of Xiaoyaosan on LPS Level of colon homogenate.

The 16s results showed that vs. the normal group, the gut microbiota of the model group mice were significantly different from the normal group at the species level (Figure 3A). Based on the results of a completely randomized multi-sample rank sum test, *Lachnospiraceae, Bacteroidaceae, Akkermansia, Lachnoclostridium, Hungatella, Robinsoniella, Alloprevotella, Prevotellaceae, Parasutterella* and other *bacterial* genera were significantly different. More important, Xiaoyaosan can significantly increase the abundance of *Lachnospiraceae*, and inhibit the growth of *Bacteroidaceae* (Figure 3B).

### Effects of XYS on the Level of LPS in Serum, Feces, Colon Homogenate and IL-1β Level of Serum in Antibiotic-Induced Mice

**FIGURE 4:**
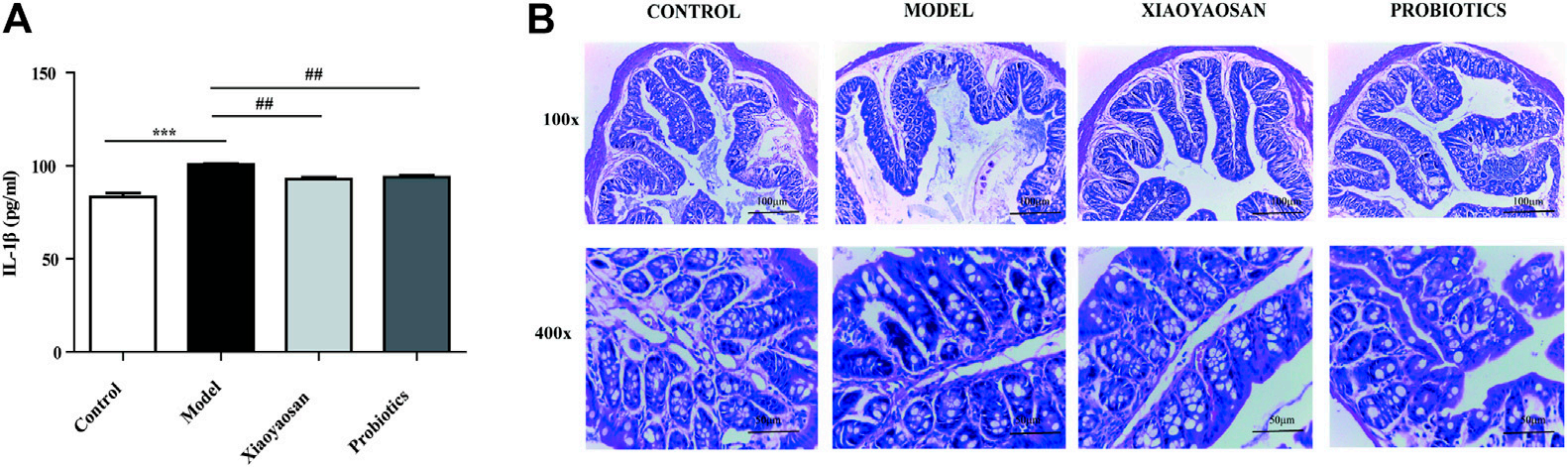
Effects of Xiaoyaosan on IL-1β and Effect of Xiaoyaosan on Colon Pathology. **(A)** The IL-1β Level of Serum. **(B)** Effect of Xiaoyaosan on Colon Pathology. Hematoxylin and eosin staining of the colon (×100magnification, ×400magnification).

To investigate the intention of Xiaoyaosan on flora-related signaling molecules, the level of LPS in serum, feces, colon homogenate was determined. As shown in Figure 3C, the level of LPS in serum in the antibiotic-induced group were higher vs. the control group (*p* < 0.001), and Xiaoyaosan and probiotic Intervention significantly lessened the release of LPS (*p* < 0.01 and *p* < 0.01, respectively) (Figures 3D,E). The same results were obtained for the level of LPS in feces and colon homogenate, indicating that after the intervention of antibiotics, there was an increase of LPS in the whole level of mice, which was significantly related to the intestinal flora, and this increase could be reversed by Xiaoyaosan and probiotics. Besides, we also determined the concentration of IL-1β in serum to observe the possible inflammatory response. As illustrated in Figure 4A, the serum IL-1β levels were remarkly higher in mice treated with antibiotic than in the control group (*p* < 0.001), and the Xiaoyaosan treatment reduced the levels (*p* < 0.01).

### Effect of XYS on Colon Pathology

As illustrated in Figure 4B, in the normal group, the colon mucosa was intact; epithelial cells were arranged neatly and there was no infiltration by inflammatory cells. In the model group, the colon mucosa was obviously, absent, the glands in the lamina propria were damaged or had disappeared, the number of goblet cells was reduced, and amount of inflammatory cells had infiltrated. In the Xiaoyaosan and probiotics group, the epithelial cell defects were not as obvious; furthermore, their basic morphology was similar to that of the normal group.

### Effects of XYS on the Expression of the NLRP3, Caspase-1 and ASC mRNAs in the Colon

**FIGURE 5:**
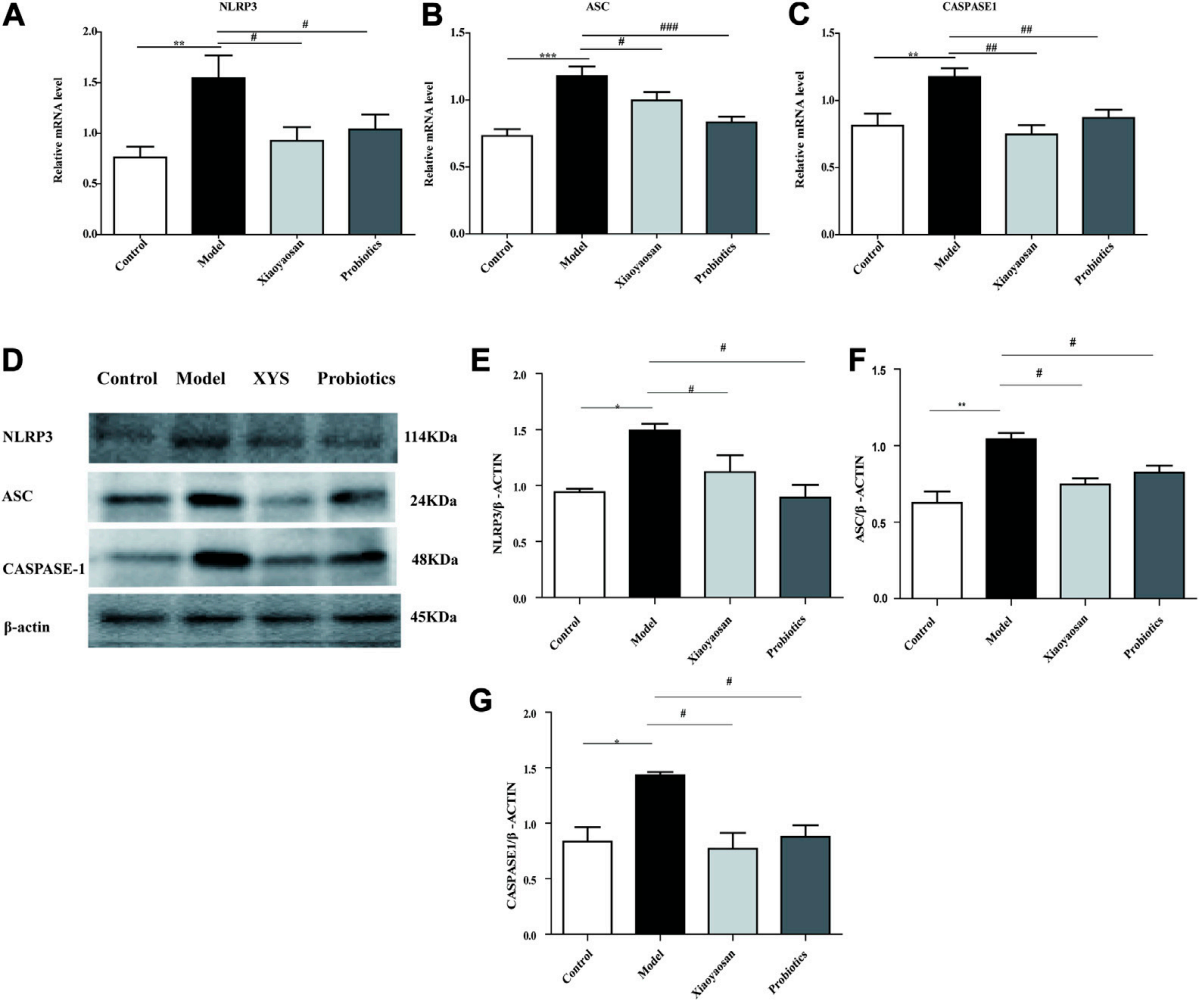
Effects of Xiaoyaosan on the NLRP3-ASC-CASPASE-1 pathway in the colon. **(A)** NLRP3mRNA in colon (*n* = 6). **(B)** ASCmRNA in colon (*n* = 6). **(C)** CASPASE-1mRNA in colon (*n* = 6). **(D)** Expression of NLRP3, ASC, CASPASE-1 protein in colon (*n* = 4). **(E)** NLRP3 protein in colon (*n* = 4). **(F)** ASC protein in colon (*n* = 4). **(G)** CASPASE-1 protein in colon (*n* = 4). Data are expressed as the mean ± SEM. *p < 0.05, **p < 0.01 vs. the control group; ^#^ *p* < 0.05, ^##^ *p* < 0.01 vs. the antibiotic-induced model group.

The relative expression levels of NLRP3, ASC and Caspase-1 genes were detected by PCR. There were significant differences in the mRNA expression of NLRP3, ASC and Caspase-1 units among groups. As illustrated in Figures 5A,B, vs. the model group, the expression of NLRP3 and ASC in the Xiaoyaosan group and the probiotics group decreased (*p* < 0.05 and *p* < 0.01, respectively). As illustrated in Figure 5C, Compared with the model group, the relative expression of the Caspase-1 gene in the Xiaoyaosan group and the probiotics group decreased significantly (*p* < 0.01).

### Effects of XYS on the Expression of the NLRP3, Caspase-1 and ASC Proteins in the Colon

As illustrated in Figure 5D,E, the expression of NLRP3 was significantly increased in colon of antibiotic-induced mice, while was down-regulated by treatment with Xiaoyaosan or probiotic (*p* < 0.05). As illustrated in Figure 5F,G, vs. the model group, the expression of the ASC and Caspase-1 in the Xiaoyaosan group and the probiotics group decreased sightly (*p* < 0.05 and *p* < 0.05, respectively), together with the previous results, indicating that Xiaoyaosan inhibit the immoderate activation of the NLRP3 inflammasome in the colon.

### Effect of XYS on Immunohistochemistry of NLRP3, Caspase-1 and ASC in Colon

**FIGURE 6:**
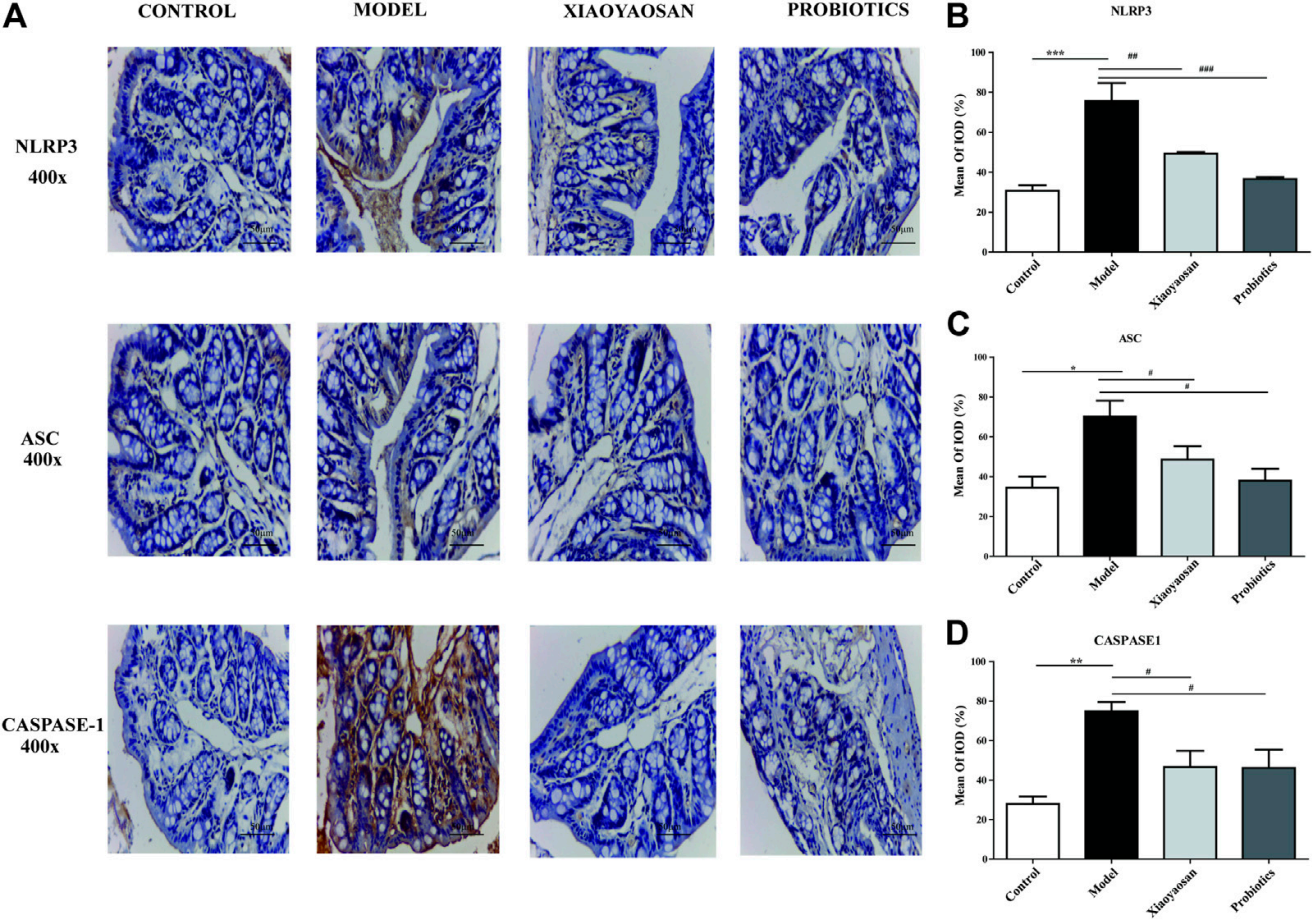
Effects of Xiaoyaosan on the NLRP3-ASC-CASPASE-1 immunolabeling in the colon. **(A)** Immunohistochemical staining of NLRP3, ASC and CASPASE-1 protein in colon (scale bar = 50 mm, ×400 magnification). **(B)** Expression. of NLRP3 in colon. **(C)** Expression of ASC in colon. **(D)** Expression of CASPASE-1 in colon. *n* = 6 per group. Data are expressed as the mean ± SEM. *p < 0.05, **p < 0.01 vs. the control group; ^#^ *p* < 0.05, ^##^ *p* < 0.01 vs. the antibiotic-induced model group.

In the model group, small brown granules representing NLRP3, Caspase-1 and ASC staining were significantly increased and strongly. For treatment of Xiaoyaosan and probiotics, the levels of NLRP3, Caspase-1 and ASC in each treatment group was significantly reduced (Figure 6A). IOD analysis showed that NLRP3 in the model group had increased significantly vs. the normal group (*p* < 0.001); Caspase-1 and ASC in the model group had increased (*p* < 0.05 and *p* < 0.01, respectively) (Figure 6B). Fxpression of NLRP3 in the Xiaoyaosan group and the probiotics group all showed significant reductions (Figures 6C,D); the levels of Caspase-1 and ASC in Xiaoyaosan group and probiotics group all showed slightly reduced levels (*p* < 0.05 and *p* < 0.05, respectively).

## Discussion

In this study, we investigated the regulatory effect of Xiaoyaosan in mouse models of depression and anxiety, using AIMD mice. The most important findings from this study are as follows: 1) Xiaoyaosan improved depressive and anxious behavior in AIMD mice. 2) Treatment with Xiaoyaosan altered the gut microbiota while reducing LPS levels in the intestinal and systemic circulation. 3) Xiaoyaosan may exert antidepressant-like and anxiolytic effects by restraining the moderate activation of the NLRP3 inflammasome in the colon.

The gut microbiota plays a significant role in the development of mental illnesses including depression and anxiety. The two-way regulation of the brain-gut axis is a vital mechanism by which gut microbiota affect the central nervous system (CNS). Through interaction between the enteric nervous system, vagus nerve, HPA axis, microbial metabolites, host signal molecules, and immune factors, gut microbiota can affect the development and regulation of CNS, achieve bottom-up regulation of CNS, and participate in the pathological processes of various mental diseases, including depression and anxiety (Rieder et al., 2017). Preclinical studies have shown that the long-term use of antibiotics can cause behavioral disorders in patients, with symptoms similar to those of depression and anxiety. Moreover, the AIMD mouse model is widely used to assess the role of the intestinal flora and efficacy of antidepressants or anxiolytics in rodents (Merzoug et al., 2002). Clinical studies have confirmed that antibiotic intervention and disturbance in intestinal flora can increase the risk of depression and anxiety (Lurie et al., 2015). The AIMD mouse model represents disorders of the intestinal flora, accompanied by behavioral changes including depression, anxiety, social disorders, and aggressive consciousness (Hao et al., 2020), and indicates a possible close relationship between intestinal flora in humans and their behavior (Leulier et al., 2017). In this study, we used an ampicillin-induced model to study anxiety and depressive behavior, and used 16S technology to determine changes in the gut microbiota. Our findings were identical to those reported in previous studies (Liang et al., 2017). We have also reproduced the antidepressant and anti-anxiety effects of Xiaoyaosan by using the chronicre straint strees and chronic unpredictable mild stress models (Guo et al., 2017), suggesting that treatment with Xiaoyaosan alters the gut microbiota, and confirming that the gut microbiota is an important target for Xiaoyaosan.

In this study, we found that Xiaoyaosan could alter microbial abundance after antibiotic intervention, reduce LPS levels, and affect intestinal inflammation, thereby providing new evidence for gut flora as a therapeutic target in the management of mental disorders. The intestinal flora plays a vital role in host metabolism and the maintenance of homeostasis of the gastrointestinal tract (Round and Mazmanian, 2009). When there is an imbalance in the gut microbiota, certain microbes can mediate intestinal metabolic imbalance and intestinal inflammation, induce metabolic diseases, and cause mental illness. *Bacteroidaceae* is a gram-negative bacterium comprising the intestinal microflora. Many clinical studies have reported a high abundance of *Bacteroidaceae* in the stools of patients with depression, anxiety, hyperlipidemia, and liver cirrhosis, suggesting that the presence of *Bacteroidaceae* may be associated with the development of mental and metabolic diseases (Xie et al., 2016). *Bacteroidaceae* are also the main producer of LPS, which is associated with the activation of inflammation and participation in a series of inflammatory diseases including colitis and hepatitis (Whitfield and Trent, 2014). In this study, we found that Xiaoyaosan could change the abundance of specific genera in the intestinal flora, which was manifested by a significant increase in the abundance of *Lachnospiraceae* and inhibition of the growth of *Bacteroidaceae*. When combined with the metagenomics and gut microbiota data of a previous experiment (Hui-Zheng et al.), we found that Xiaoyaosan could significantly reduce the level of LPS in mouse stools, colon homogenate, and serum. These findings demonstrated that abnormal LPS levels in the intestinal and peripheral circulation were corrected after the restoration of the intestinal flora. This study also revealed a relationship between LPS and colon inflammation. AIMD mice exhibited significant colonic inflammation and increased LPS levels, which were markedly reduced after treatment with Xiaoyaosan, resulting in an improved inflammatory status of the colon. This result corroborates previous findings and highlights the role of LPS in inflammation. To summarize, our findings suggested that the reduction of LPS and the improvement of colon inflammation mediate the antidepressant and anxiolytic effects of Xiaoyaosan.

We found that Xiaoyaosan could influence antibiotic-induced depressive behavior by inhibiting NLRP3 inflammasome activation in the colon. The NLRP3 inflammasome can be activated by LPS to induce caspase-1 cleavage, leading to the maturation of the pro-inflammatory cytokines IL-1β and IL-18 (Ye et al., 2016). Several studies have demonstrated that the NLRP3 inflammasome is involved in the pathogenesis of depression and anxiety (Alcocer-Gómez and Cordero, 2014). Activation of NLRP3 and IL-1β is observed in patients with depression and anxiety (Alcocer-Gómez et al., 2016). Thus, targeting the NLRP3 inflammasome may be a new approach for treating depression (Du et al., 2016). In this study, we used AIMD mice and found that ampicillin activated the NLRP3 inflammasome in the colon and released a large amount of IL-1β in the colon and serum. Xiaoyaosan treatment abolished the antibiotic-induced activation of the NLRP3 inflammasome and improved depressive and anxious behavior, as evidenced by a striking increase in the total distance in the OFT and a decrease in the duration of immobility. This evidence indicates that inhibiting NLRP3 inflammasome activation may result in antidepressant and anxiolytic effects similar to those observed in Xiaoyaosan-treated mice.

The two-way regulatory effect of Xiaoyaosan on depression and anxiety has been previously reported. For example, Xiaoyaosan can exert anxiolytic effects through the CRF1 receptor (Jiang et al., 2016) and the JNK signaling pathway (Zhao et al., 2017) and can improve depressive behavior by targeting the Nesfatin-1 receptor (Ma et al., 2019). Our current research confirmed the two-way regulation of anxiety and depression by Xiaoyaosan could be attributed to the alteration in the abundance of intestinal flora and reduction in endotoxin-induced inflammation.

Our study has some limitations. First, Xiaoyaosan contains multiple Chinese herbal medicines, and its ingredients are complex; we have only demonstrated its antidepressant and anxiolytic effects in combination (Gao et al., 2018). The activity and synergy between components are not yet clear, and further studies are needed to elucidate the component or components responsible for the effects we have observed. Second, the results of our study suggest that the levels of LPS and inflammatory factors in the systemic circulation increase but whether this directly affects the brain through the blood-brain barrier has not been addressed. More importantly, the direct evidence that Xiaoyaosan improves depression-like behaviors through intestinal flora needs to be further studied. Therefore, in future work, we will use fecal microbiota transplantation (FMT) and germ-free mice (GF) to further study the antidepressant and anti-anxiety effects of Xiaoyaosan based on intestinal microbes. We plan to focus on this aspect in our future research.

## Conclusion

**FIGURE 7:**
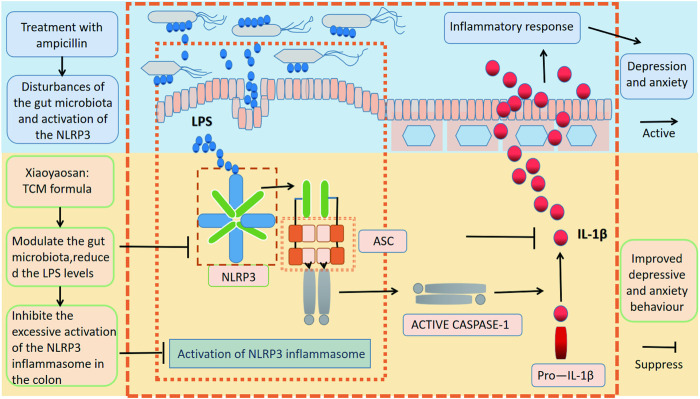
Xiaoyaosan suppresses anxiolytic-like behaviors and improve depressive behavior by modulating the gut microbiota and inhibiting the excessive activation of the NLRP3 inflammasome in the colon.

We have demonstrated a potential role of the gut microbiota in the antidepressant and anxiolytic effects of Xiaoyaosan in the AIMD mouse model (Figure 7), thereby enriching our understanding of the pharmacology of Xiaoyaosan. This innovative research has revealed the antidepressant and anti-anxiety effects of Xiaoyaosan through its effect on endotoxin metabolism disorder caused by intestinal flora and the excessive activation of the NLRP3 inflammasome. Our findings suggest that Xiaoyaosan may improve anxiolytic and depressive behavior by modulating the gut microbiota and inhibiting the immoderate activation of the NLRP3 inflammasome in the colon, offering further evidence for the clinical application of Xiaoyaosan.

## Data Availability

The datasets presented in this study can be found in online repositories. The names of the repository/repositories and accession number(s) can be found below: NCBI SRA BioProject, accession no: PRJNA716118.
